# Longitudinal Trends in Hypertension Management and Mortality Among Octogenarians: Prospective Cohort Study

**DOI:** 10.1161/HYPERTENSIONAHA.116.07246

**Published:** 2016-06-08

**Authors:** Alex Dregan, Rathi Ravindrarajah, Nisha Hazra, Shota Hamada, Stephen H.D. Jackson, Martin C. Gulliford

**Affiliations:** From the Department of Primary Care and Public Health (A.D., R.R., N.H., S.H., M.C.G.), National Institute for Health Research Biomedical Research Centre at Guy’s and St. Thomas’ National Health Service Foundation Trust (A.D., M.C.G.), and Department of Clinical Gerontology (S.H.D.J.), King’s College London, London, United Kingdom.

**Keywords:** aging, blood pressure, hypertension, mortality, prevalence

## Abstract

The role of hypertension management among octogenarians is controversial. In this long-term follow-up (>10 years) study, we estimated trends in hypertension prevalence, awareness, treatment, and control among octogenarians, and evaluated the relationship of systolic blood pressure (SBP) ranges with mortality. Data were based on the English Longitudinal Study of Ageing (ELSA). Outcome measures were hypertension prevalence, awareness, treatment and control, and cardiovascular disease, and all-cause mortality events. Participants were separated into 8 categories of SBP values (<110, 110–119, 120–129, 130–139, 140–149, 150–159, 160–169, and >169 mm Hg). Among 2692 octogenarians, mean SBP levels declined from 147 mm Hg in 1998/2000 to 134 mm Hg in 2012/2013. The decline was of lower magnitude in the 50 to 79 years old subgroup (n=22007). Hypertension prevalence and awareness were 40% and 13%, respectively, higher among octogenarians than the 50 to 79 years of age subgroup, but hypertension treatment rates were similar (≈90%). Around 47% of the treated octogenarians achieved conventional BP targets (<140/90 mm Hg), increasing to 59% when assessed against revised targets (<150/90 mm Hg). All-cause mortality rates were higher (hazard ratio, 1.55; 95% confidence interval, 0.89–2.72) at lower extremes of SBP values (<110 mm Hg). The lowest cardiovascular disease and all-cause mortality risk among treated octogenarians was observed for an SBP range of 140 to 149 mm Hg (1.04, 0.60–1.78) and 160 to 169 mm Hg (0.78, 0.51–1.21). An increasing trend in hypertension awareness and treatment was observed in a large sample of community-dwelling octogenarians. The results do not support the view that more stringent BP targets may be associated with lower mortality.

## Abstract 2

Supplemental Digital Content is available in the text.

Cardiovascular diseases (CVDs) are responsible for almost 17 million deaths worldwide, with hypertension being a major contributor to over half of these cases (55%).^1^ Two thirds (66%) of deaths among people >85 years of age are CVD related, and this age group presents high rates of hypertension. Hypertension is associated with increased risks of coronary heart disease, stroke, peripheral vascular disease, cognitive impairment, renal impairment, and visual impairment among others.^2–4^ Although the benefits of lowering elevated blood pressure (BP) levels are conclusive among younger population groups,^5^ there is an ongoing debate about the benefits of treatment and optimal therapeutic BP target among octogenarians. Evidence for a negative association between BP with mortality and CVD events supports the view that lower BP may be associated with better outcomes.^5–8^ There is disagreement about how low the target BP should be set among octogenarians, with clinical trials suggesting different systolic BP (SBP) targets, including <120,^9^ <130,^5^ <140,^10^ or <150 mm Hg.^11^ Other authors^12,13^ suggested that among octogenarians both lower and higher values can lead to adverse events. Consistent with this evidence, current guidelines favor a more relaxed therapeutic target for BP in octogenarians (SBP <150 mm Hg).^14,15^ This ongoing disagreement about hypertension management in very old people underlines the need for further investigations to ascertain optimal BP levels in community-living octogenarians, the population subgroup in whom hypertension is most prevalent. Thus, the first objective of this study was to estimate the relationship of SBP ranges with mortality among octogenarians. Given recent cross-sectional evidence about underdiagnosis of hypertension in community populations,^16^ the second objective of this study was to evaluate trends in hypertension prevalence, awareness, treatment, and control among people younger than and older than 80 years of age.

## Methods

The study uses data from the English Longitudinal Study of Ageing (ELSA), a prospective study of a nationally representative sample of adults aged ≥50 years living in private households in England. The ELSA sample was drawn from 3 years (1998, 1999, and 2001) of the Health Survey for England (HSE) survey (wave 0). The original ELSA sample was followed-up biannually, including 2002/2003 (wave 1), 2004/2005 (wave 2), 2006/2007 (wave 3), 2008/2009 (wave 4), 2010/2011 (wave 5), and 2012/2013 (wave 6). A broad range of health, demographic, socioeconomic, and lifestyle data were collected at each survey, including clinical measurements carried out by trained nurses every 4 years (wave 0, wave 2, wave 4, and wave 6). At waves 3 and 4, the study was replenished with new study participants from the HSE to maintain the size and representativeness of the study. This approach accounts for variation in eligible participants included in the analyses across different waves. A detailed description of methods, response rates, and sampling procedures can be found elsewhere.^4,17,18^ The analytic sample for this study included 24 699 participants aged ≥50 years at wave 0 and who were eligible to be included in the ELSA study. Participants gave their consent to participate in the study and ethical approval was granted from the London Multicentre Research Ethics Committee.

### Outcome Measures

Mortality outcomes were all-cause mortality and CVD-related mortality. These measures were based on mortality data up to February 2012, the latest date when the ELSA study mortality data were updated from the National Health Service Central Data Registry (NHSCR) records. NHSCR contains computerized records of all NHS patients, including reliable data of everyone who has died in England. ELSA also records NHSCR data on participants’ main cause of death (using *International Classification of Diseases Ninth Revision* and *International Classification of Diseases Tenth Revision* coding), which was used to develop the CVD-related outcome measure. A small number of participants who did not give permission to check NHSCR records (n=458), but who were identified as being deceased from other sources (fieldwork or next of kin) were included in the analysis.

### Exposure Variables

#### BP Measurement

All participants were eligible to have their BP measured. Three separate BP readings were taken 1 minute apart on seating participants, by the nurse using the Omron HEM-907 monitor (Dinamap at baseline). BP data from baseline were Omron adjusted to allow comparability with following years using previous equations.^19^ The study used the mean of the second and third SBP and diastolic BP (DBP) readings. To avoid regression to the mean bias from using only baseline BP values, and following Lewington et al’s^20^ suggestions, the study calculated the average BP across baseline (wave 0), wave 2, and wave 4 data. Wave 6 data were not used in the main analyses because of the lack of updated mortality data beyond wave 5. SBP measurements were then used to classify participants into 8 groups in increments of 10 mm Hg (<110, 110–119, 120–129, 130–139, 140–149, 150–159, 160–169, and ≥170 mm Hg). DBP values were used to classify participants into 7 groups with an increment of 10 mm Hg (<50, 50–59, 60–69, 70–79, 80–89, 90–99, and ≥100 mm Hg).

#### Hypertension Prevalence, Treatment, Monitoring, and Control

For comparability with recent US-based evidence^21^ within a UK context, the study explored trends in hypertension prevalence, awareness, treatment, and control. Participants were considered as hypertensive if they had SBP ≥140 mm Hg and DBP ≥90 mm Hg, reported a previous physician-diagnosed hypertension, and were current users of antihypertensive medication. Participants reporting a previous physician-diagnosed hypertension or if they reported current taking of antihypertensive medication were categorized as being aware of having hypertension. Participants who were on current antihypertensive medication were categorized as a treatment group. Finally, 2 BP-controlled hypertension categories were developed: (1) SBP/DBP of <150/90 mm Hg and (2) SBP/DBP of <140/90 mm Hg reflecting Eight Joint National Committee (JNC-8) and, respectively, JNC-7 treatment recommendations. In addition, a self-reported BP monitoring variable was included to reflect the proportion of hypertensive people with a BP consultation in the past 12 months.

### Covariates

Several factors known to be associated with hypertension and mortality were included as covariates. Age was included as a continuous variable and sex as a binary variable. Smoking classified participants into never, ex-smokers, and current smokers. Participants were classified into underweight (body mass index [BMI], <18.5 kg/m^2^), optimal (BMI, 18.5–24.9 kg/m^2^), overweight (BMI, 25–29.9 kg/m^2^), and obese (BMI ≥30 kg/m^2^), according to their baseline BMI. Data about the type and amount of physical activity participation were used to group participants into sedentary or low and moderate or vigorous activity. Social class was defined as a binary variable, grouping participants into manual and nonmanual occupations. Long-standing illness (including CVD, type 2 diabetes mellitus, arthritis, cancer, liver disease, and chronic kidney disease) was included as a binary variable. Depression has been associated with increased hypertension risk,^22^ and it was assessed as a continuous variable using the 12-item General Health Questionnaire. Total cholesterol value was included as a continuous variable. Finally, antihypertensive medication intake was included as a binary variable. C-reactive protein and frailty measures were also considered initially but excluded from final analyses as they did not influence the association between SBP with mortality.

### Statistical Analysis

Study sample characteristics were analyzed using descriptive statistics. Descriptive statistics were used to denote the prevalence of hypertension and the proportion of people with hypertension awareness, treated, monitored, and controlled. Participants contributed follow-up time (person-years) from the time of entry into the study until the date of death, or the end date of the study or the date of last known contact. Cox proportional hazard models were used to estimate the association between BP categories with all-cause mortality adjusting for study covariates. Separate analyses were conducted for hypertension-treated participants, those aged ≥80 years, and the full sample. Because non-CVD mortality can be considered a competing event for CVD-related mortality, competing risks regression analyses were used to estimate hazard ratios and 95% confidence intervals of CVD-related mortality for categories of SBP and DBP. In all analyses, the reference group for SBP was 120 to 129 mm Hg and for DBP it was 70 to 79 mm Hg. An SBP range of 120 to 129 mm Hg is considered optimal in adult population,^23^ and it has been chosen as the reference category in our study. Consistent with previous studies,^24,25^ the nadir range in our study was defined as the SBP range associated with the lowest mortality estimates, after which lowest values tended to be associated with higher mortality rates. To minimize the potential for reverse causality bias, the analyses excluded participants who died within 6 months from study baseline and within 60 days^26^ from a follow-up BP measurement (n=209). The hazards proportionality assumption was tested using Schoenfeld residuals against survival time, which revealed no violation of this assumption. Models were adjusted for age, sex, BMI, long-standing illness, cholesterol values, BP treatment, smoking, physical activity, depression, DBP, and social class. The decision to adjust for DBP was based on suggestions that the relation of SBP and CVD mortality varies with DBP values.^27^ Multiple imputation with chained equations was used to handle missing data using 10 imputed data sets and including all study variables in the imputation model. Planned sensitivity analyses were conducted that included the exclusion of patients with CVD diagnosis at baseline, and the use of restricted SBP categories (<120, 120–129, 130–139, 140–149, 150–159, and >160 mm Hg). Following the study by Aparicio et al,^28^ we also used Cox regression to estimate mortality risk associated with BP expressed as a continuous predictor. We evaluated whether there was a deviation from linearity using the Wald test for nonlinear hypotheses and, if present, estimated whether a quadratic term improved goodness-of-fit. In all analyses, a 2-sided *P*<0.05 was chosen as the criterion for statistical significance. All analyses were carried out using STATA version 13. Because SBP is a stronger predictor of mortality risk compared with DBP in people >60 years of age,^29^ only the results for SBP are discussed here (DBP results are available from the authors). The study presents the results for patients aged 50 to 79 years for comparative purposes only, with the main focus being on the results for octogenarians.

## Results

The ELSA included 24 699 participants with a mean of 7 (range, 0–15) years as follow-up years. Table 1 shows the characteristics of the participants at baseline by 10 mm Hg SBP categories. Mean age was greater for higher SBP values. Participants with highest SBP values were generally older, presented higher rates of chronic illness, and were more likely to be obese, from poor social background, and physically inactive. Participants with the lowest SBP levels were more likely to be female, younger, underweight, and present higher rates of smoking and depressive symptoms. The amount of missing data in the analyses varied from around 6% (ie, social class) to 42% (ie, physical activity levels).

**Table 1.**
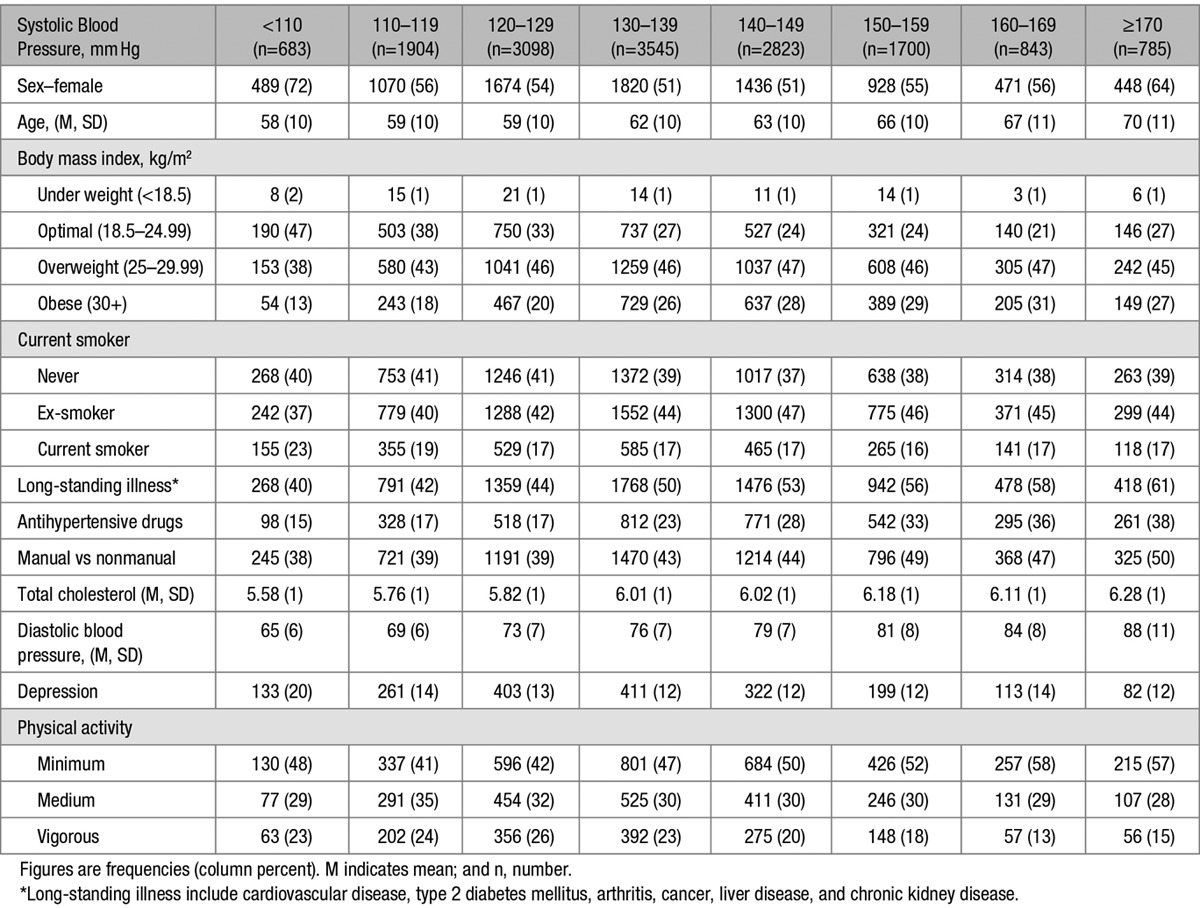
Study Population Characteristics at Baseline According to Hypertension Subtype

Table 2 shows the longitudinal trends in awareness, treatment, and control of hypertension in all study participants, and those aged ≥80 years, contrasted with those aged <80 years. Rates of awareness of hypertension, treatment of hypertension, as well as the proportion of treated participants who achieved recommended SBP targets generally increased over time. Similar trends emerged when participants were divided into ages <80 years and ≥80 years, with the latter presenting a steady improvement in all domains during the study period. Although a higher proportion of people aged ≥80 years were aware of being hypertensive and were on treatment compared with those aged <80 years, a lower proportion achieved recommended BP targets (<150/90 mm Hg).

**Table 2.**
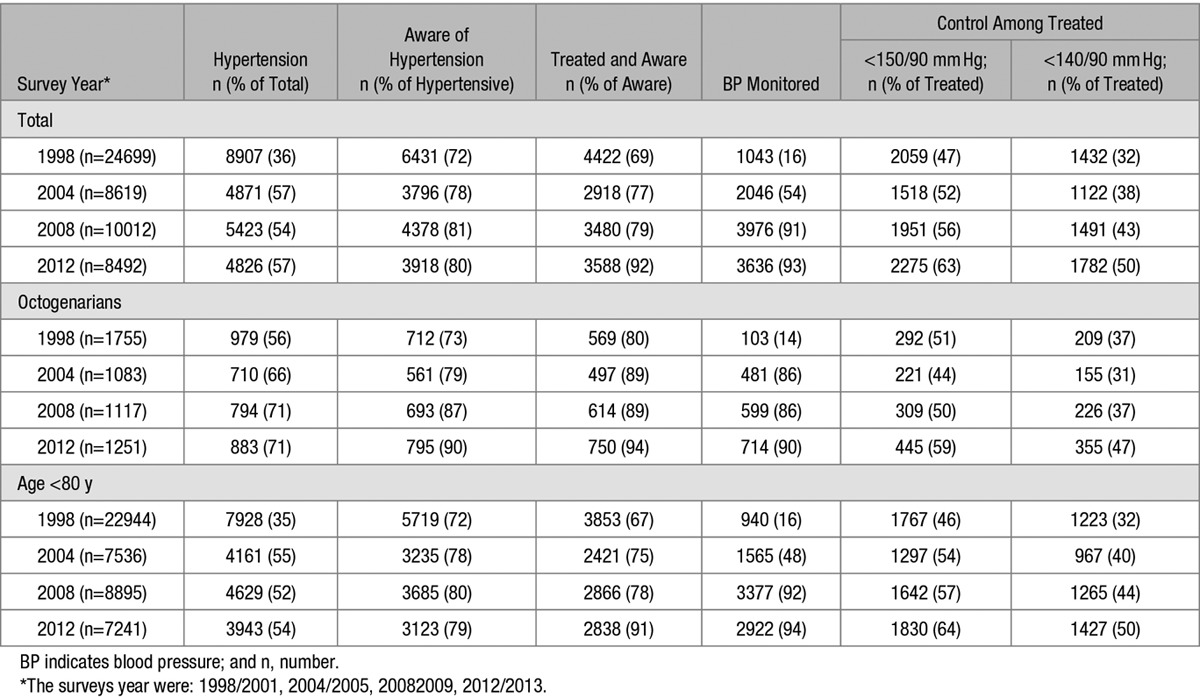
Longitudinal Trends in Self-Reported Awareness, Treatment, Monitoring, and Control of Hypertension From 1998/2001 to 2012/2013

**Figure 1.**
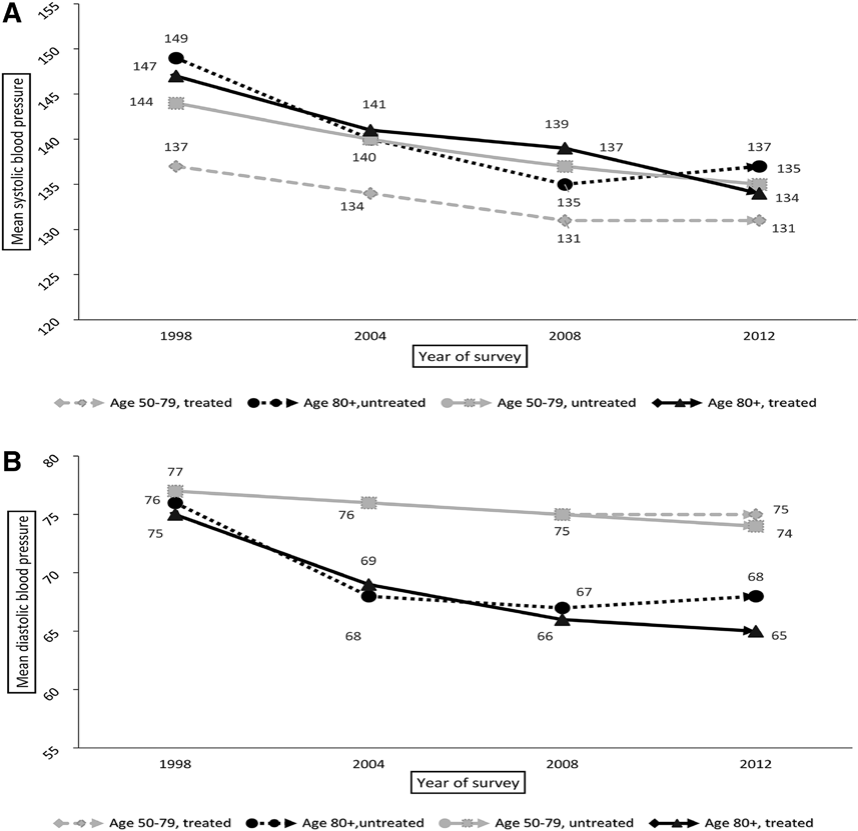
Patterns of longitudinal changes in mean systolic (**A**) and diastolic (**B**) blood pressures.

Patterns of longitudinal changes in mean BP levels for treated and untreated participants are illustrated in Figure 1. A declining trend was observed in mean SBP among treated octogenarians from 147 mm Hg in 1998/2001 to 134 mm Hg in 2012/2013. Thus, there was an absolute difference in mean SBP >12 years period of −12 mm Hg (95% confidence interval, −15 to −9; *P*<0.001). Untreated octogenarians showed an initial decline in mean SBP (135 mm Hg) to 2008/2009, followed by a modest increase in mean SBP (137 mm Hg) to 2012/2013.

**Figure 2.**
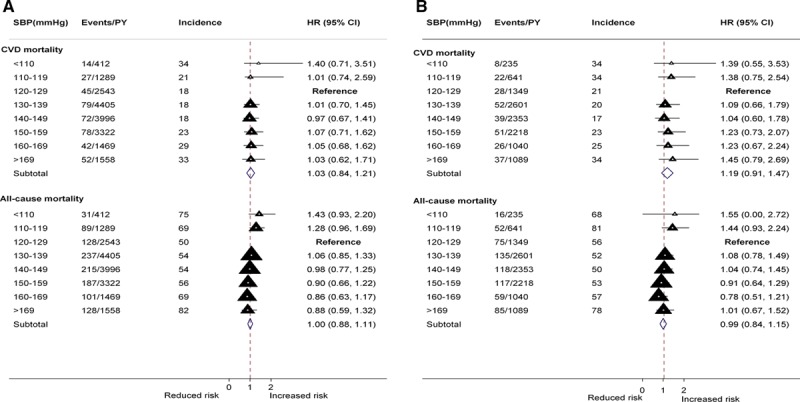
Adjusted hazard ratios (HR) for mortality by systolic blood pressure (SBP) levels among total (**A**, n=2692) and treated (**B**, n=1299) octogenarians. CI indicates confidence interval.

The results of Cox regression analyses for octogenarians are presented in Figure 2. Although not statistically significant, adjusted competing risks analyses among treated older participants suggested that the association between SBP and CVD mortality might follow a J-shaped curve, with increased point estimates for both low and high extremes of SBP. The lowest event rate SBP range was between 140 and 149 mm Hg (hazard ratio, 1.04; 95% confidence interval, 0.60–1.78). The association of SBP with all-cause mortality seemed to follow an inverse J-shaped curve with the critical nadir SBP range between 160 and 169 mm Hg (0.78, 0.51–1.21).

**Figure 3.**
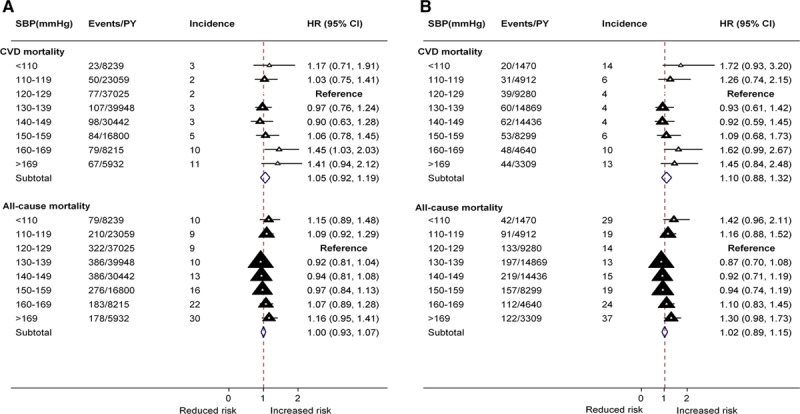
Adjusted hazard ratios (HR) for mortality by systolic blood pressure (SBP) levels among total (**A**, n=22007) and treated (**B**, n=6311) people aged 50 to 79 years.

Among participants aged 50 to 79 years (Figure 3), the association of SBP with both CVD and all-cause mortality followed a J-shaped curve. The critical nadir SBP range for CVD and all-cause mortality risk among treated participants was between 140 and 149 mm Hg and 130 and 139 mm Hg, respectively. Similar patterns were revealed in the combined treated and untreated analyses. None of the associations reached statistical significance level.

### Sensitivity Analyses

Sensitivity analyses that excluded patients with CVD diagnosis at baseline and that used restricted SBP categories validated the patterns of the association between SBP with mortality outcomes in primary analyses (Figures S1–S4 in the online-only Data Supplement). Analyses based on continuous BP measures (Table 3) identified that a model incorporating a quadratic term yielded improved goodness-of-fit than a linear model among treated octogenarians (*P*<0.001). These analyses revealed a modestly significant association between all-cause mortality with SBP (β, −0.04, −0.09 to −0.00) among treated octogenarians. Because the interpretation of coefficients from quadratic regression do not have a straightforward interpretation, supplementary plots are provided in Figure S4. These plots suggest that the association between SBP with all-cause mortality among treated octogenarians is U-shaped with the nadir at 167 mm Hg. A similar trend was observed for the 50 to 79 years of age subgroup with the nadir at 143 mm Hg.

**Table 3.**
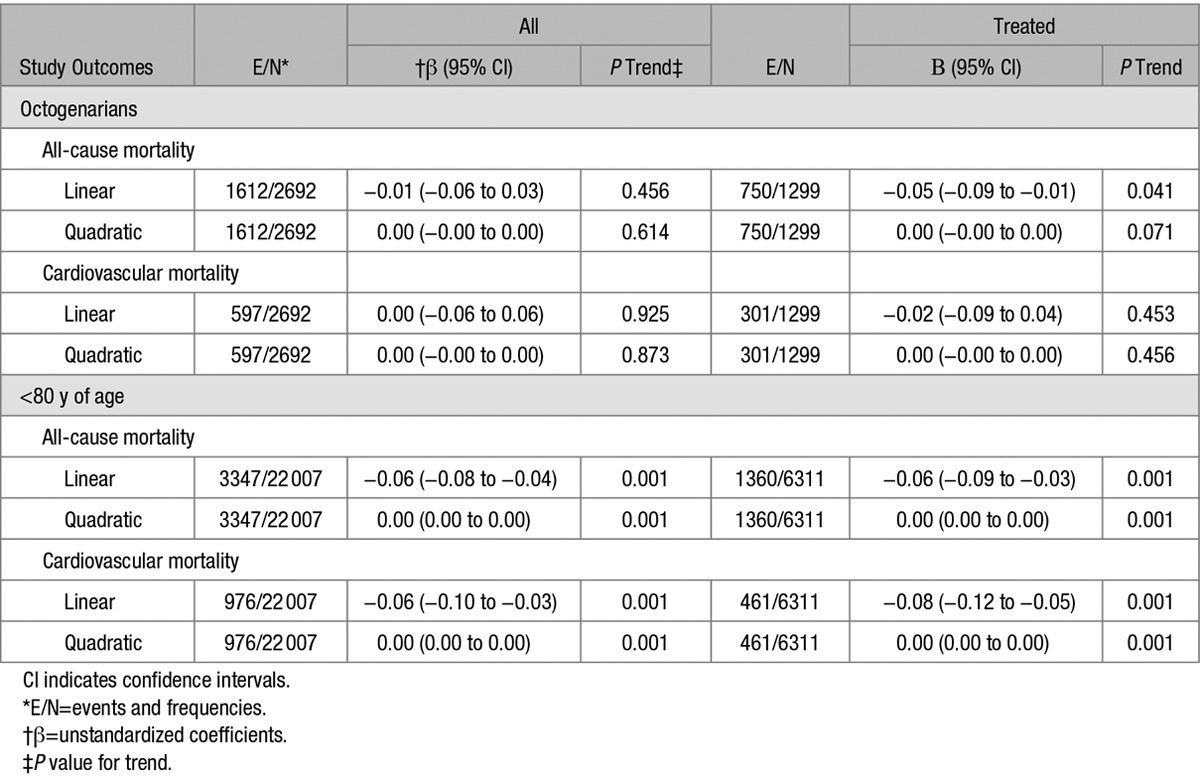
Adjusted Unstandardized Coefficients Relating Continuous Systolic Blood Pressure Measure to All-Cause and Cardiovascular Mortality

## Discussion

In this 12-year prospective study, the mean SBP among octogenarians decreased by 13 mm Hg between 1998/2001 and 2012/2013. A similar trend of lower magnitude (7 mm Hg) was observed among the 50 to 79 years old participants. After an initial increment from baseline to 2004, the prevalence of hypertension remained constant at around 70% among treated octogenarians, compared with around 50% in the 50- to 79-year-old group. Hypertension awareness rates were higher among octogenarians compared with those aged 50 to 79 years; however, hypertension treatment and BP monitoring rates were similar. Using the BP target of <150/90 mm Hg, around 41% of treated octogenarians would be classified as having uncontrolled hypertension, a 23% lower figure compared with the target of <140/90 mm Hg suggested by traditional guidelines. One possible explanation for the high proportion of octogenarians with uncontrolled hypertension is that in deciding whether to lower an older patient’s BP level a clinician may rely on multiple health indicators, knowledge about the patient’s BP history and their own clinical experience. Also, faced with differing empirical evidence clinicians may be reluctant to set specific BP targets for very old participants. Although not statistically significant, all-cause mortality tended to be higher at low-SBP ranges, and CVD mortality was higher at both extreme low- and high-SBP ranges. Thus, our findings are suggestive of a J-curve association between SBP with all-cause mortality among community-living octogenarians, although conventional significance levels were not reached. This suggestion is supported by the nonlinear association observed between continuous SBP measures with all-cause mortality among treated octogenarians. Our study identified an association of SBP with CVD mortality among the 50 to 79 years old participants mainly at higher extremes of SBP.

The lack of significant association between SBP and mortality among treated octogenarians may suggest a beneficial role for antihypertensive therapy in survival.^30^ It is also possible that the study was insufficiently powered to allow detection of small variations in mortality events at the extreme levels of SBP among octogenarians. It might also be possible that among octogenarians differences in mortality events may become important at even higher (ie, >185 mm Hg) or lower (<100 mm Hg) SBP cut points than we evaluated.^31^ These suggestions underline the importance of further prospective studies with larger populations of octogenarians to verify and expand this study findings. The nonlinear relationship between SBP with all-cause mortality challenges the view that lower SBP values will generally be associated with better outcomes^6^ and supports recent suggestions that lower BP is not necessarily better among octogenarians.^32^ In accord with recent guidelines for less aggressive BP treatment target in octogenarians,^14^ the lowest mortality rates in treated octogenarians emerged for SBP ranges of 140 to 149 mm Hg (CVD) and 160 to 169 mm Hg (all-cause mortality).

Longitudinal changes in hypertension prevalence, awareness, treatment, and control among community-living octogenarians are not well described, but the trends for the 50 to 79 years of age people are similar to findings based on earlier cross-sectional studies.^21,33–35^ Evidence about BP monitoring trends among community-living octogenarians is scarce, and this study findings imply improved practice because the introduction of the Quality and Outcomes Framework in April 2004.The suggestion of an inverse J-curve association between SBP with all-cause mortality is supportive of earlier longitudinal studies with octogenarians.^36,37^ Studies^38,39^ with younger populations suggested increased all-cause mortality associated with high-SBP ranges. Our study confirms these findings for the 50 to 79 years old people, but not among octogenarians implying age different prognostic outcomes associated with similar SBP ranges. In a Finnish cohort of people >85 years of age, Matilla et al^13^ found increased mortality rates associated with low-SBP and lower mortality rates for SBP values >160 mm Hg, as suggested by our study performed in a UK context. A meta-analysis of randomized trials^12^ found no association between hypertension treatment and all-cause mortality in octogenarians. Our study findings of no significant association between SBP levels with mortality among octogenarians seem to support this evidence. The Hypertension in the Very Elderly Trial (HYVET) recommended an SBP target of 150/80 mm Hg among treated older people,^11^ which is supported by this study evidence that among treated octogenarians the nadir SBP range for CVD mortality was around 140 to 149 mm Hg. A recent clinical trial^9^ suggested beneficial effects of lower SBP (<120 mm Hg) on mortality in patients >50 years of age and at high risk of CVD. Our findings based on a more representative sample of community-dwelling older people cautions against lowering SBP levels <110 mm Hg among octogenarians.

### Strengths and Weaknesses

This study has several strengths including prospective design, national representative sample, multiple SBP ranges, and validated mortality data. All study participants had their BP measured with a similar instrument and time frame, and the values were averaged over multiple time points reducing the risk of residual dilution bias.^40^ As with most observational data, there are also important shortcomings. Reverse causality is common in observational designs, and we cannot exclude the possibility that undetected disease at baseline may partially account for the findings. This study minimized this bias by adjusting for chronic illness at baseline and by excluding events within 6 months from study start date or within 60 days from BP measurements. ELSA study participants are well characterized in general which allowed us to adjust for important covariates; however, residual confounding (ie, patient choice and healthcare quality) remains a possibility. Selection and confounding by indication are other sources of concern. The longitudinal nature of this study ensured that people with both lower and higher BP levels, as well as treated and untreated hypertension, were included in the study and followed-up over time. Moreover, the analyses adjusted for both antihypertensive treatment and baseline CVD, further minimizing the possibility of confounding by indication.^41^ The study also used multiple imputation to impute values for participants with missing data, which further minimized the impact of selection and attrition bias on study findings. The small number of events toward the extremes of BP values may have possibly resulted in insufficient power to determine precise estimates for mortality risk among these groups. Additional analyses that reduced the number of SBP subgroups (ie, <120, 120–129, 130–139, 140–149, 150–159, and >159 mm Hg) validated, however, the study’s main findings (data available from the authors). The study findings also need to be considered in the context of an arbitrary reference category, and deviation from overall population mean has been suggested as an alternative.^28^ Using deviation from population mean modeling with our data revealed similar patterns of association to the primary analyses (data available from the authors). As Hosmer et al^42^ suggested that the interpretation of the estimated coefficients from deviations from mean coding is not as easy or clear as when reference cell coding is used, we included the findings based on the latter modeling here. Although participants were requested to show the label of the medicines taken to the interviewer, we cannot exclude the possibility for poor medication adherence, and no information was collected on the treatment regimens (ie, dosage, type, and duration). As our study participants’ BP was assessed in their own home, white coat effect or masked hypertension concerns are possibly minimal here.

In conclusion, we identified improving trends in hypertension awareness, treatment, and BP monitoring with age. Although the increment in the proportion of treated octogenarians with controlled hypertension was encouraging, a substantial number had uncontrolled hypertension. The declining trend in mean SBP values in people younger than and older than 80 years of age may reflect both improved hypertension management and positive lifestyle behavioral changes. The latter supports the value of evaluating a potential role of lifestyle interventions for hypertension management among octogenarians. The apparent sharp increase in mortality rates associated with SBP ranges <110 and ≥170 mm Hg, support a wider range of SBP targets and treatment initiation for octogenarians. Although our findings corroborate with recent clinical recommendations for a more flexible approach to BP management among older people, they need confirmation with routinely collected data and randomized trials. The lack of statistical association may reflect a more restricted role for BP on mortality in people who survive into old age or inadequate study power warranting future investigations with larger samples.

### Perspectives

In summary, our findings imply significant progression in hypertension management and monitoring among octogenarians. These improvements seem to coincide with the introduction of Quality and Outcomes Framework program, supporting the value of similar public health policies targeting very old people. The study findings support a more individualized approach to treatment initiation and optimal BP targets among octogenarians. The challenges of implementing clinical trials to confirm these findings may be overcome by the use of routinely collected primary care data that possess greater external validity. Future studies are needed to also consider clinical (ie, heart disease and stroke) and patient-centred outcomes (ie, quality of life and functioning) associated with different SBP targets.

**Figure 4.**
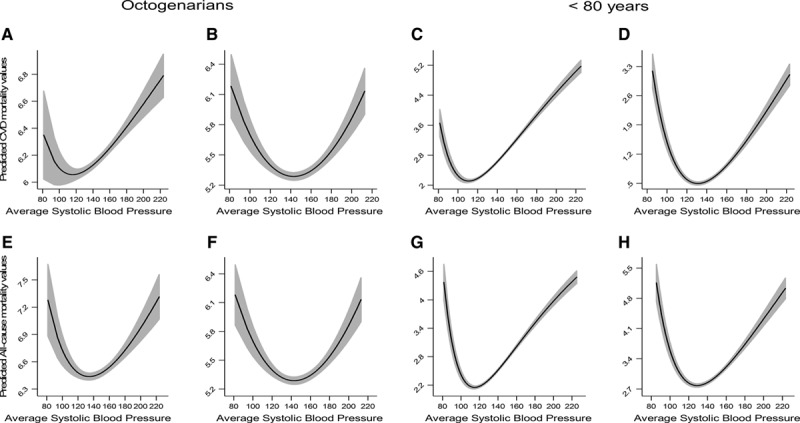
Fractional polynomial plots presenting the quadratic relationship between continuous systolic blood pressure (SBP) with total (**A**) and treated (**B**) cardiovascular disease (CVD) mortality and total (**E**) and treated (**F**) all-cause mortality among octogenarians (**left**) and the quadratic relationship between continuous SBP with total (**C**) and treated (**D**) CVD mortality and total (**G**) and treated (**H**) all-cause mortality among participants <80 years of age (**right**).

## Acknowledgments

A. Dregan and M.C. Gulliford are supported by the National Institute for Health Research Biomedical Research Center at Guy’s and St. Thomas’ National Health Service Foundation Trust and King’s College London. The views expressed are those of the author(s) and not necessarily those of the National Health Service, the National Institute of Health Research, or the Department of Health.

## Sources of Funding

This work was supported by the Dunhill Medical Trust (research grant R392/1114).

## Disclosures

None.

## Supplementary Material

## Novelty and Significance

### What Is New?

- First prospective study >10 years focusing on community-dwelling octogenarians.
- New findings about mortality risk associated with different systolic blood pressure categories.

### What Is Relevant?

- Despite improved hypertension management, a large proportion of treated octogenarians have uncontrolled hypertension.
- All-cause mortality events among octogenarians increased with low but not high extremes of systolic blood pressure.
- Among treated octogenarians, the association between systolic blood pressure with all-cause mortality is better described by a nonlinear function.

### Summary

Hypertension treatment, awareness, and monitoring improved among octogenarians to a greater extent than for those aged 50 to 79 years. The findings propose a more relaxed approach to blood pressure targets among octogenarians, and warn against systolic blood pressure values <110 mm Hg.
