# Supplementary material for: Longitudinal Trends in Hypertension Management and Mortality Among Octogenarians: Prospective Cohort Study
Source: Hypertension. 2016 Jun 8;68(1):97–105. doi: 10.1161/HYPERTENSIONAHA.116.07246 (PMC4900418; doi:10.1161/HYPERTENSIONAHA.116.07246)
Supplement: Supplementary file 1 [file hyp-68-97-s001.doc]

**On-line Data Supplement**

**Title:** Longitudinal trends in hypertension management and mortality among octogenarians. Prospective cohort study

**Authors:** Alex Dregan, Rathi Ravindrarajah, Nisha Hazra, Shota Hamada, Stephen HD Jackson, Martin C Gulliford

**Correspondence:** Dr Alex Dregan, Department of Primary Care and Public Health, 5th Floor, Addison House, Guy’s Campus, London, SE1 1UL, UK. Tel: 0207 8486639, Email: alexandru.dregan@kcl.ac.uk.

**Contents:**

Supplementary Figure S1

Supplementary Figure S2

Supplementary Figure S3

Supplementary Figure S4

**

**
